# Enhancing the Therapeutic Profile of Dapagliflozin: Chitosan Nanoparticle Encapsulation for Intestinal Applications

**DOI:** 10.1096/fba.2026-00050

**Published:** 2026-05-14

**Authors:** Agni Klonari, Antrea‐Maria Athinodorou, Eirini Papanikolaou, Anastasia Skonta, Myrto G. Bellou, Lampros Lakkas, Konstantinos I. Tsamis, Georgios S. Markopoulos, Haralambos Stamatis, Petros Bozidis, Dimitrios Peschos, Yannis V. Simos

**Affiliations:** ^1^ Department of Physiology, Faculty of Medicine, School of Health Sciences University of Ioannina Ioannina Greece; ^2^ Nanomedicine and Nanobiotechnology Research Group University of Ioannina Ioannina Greece; ^3^ Laboratory of Biotechnology, Department of Biological Applications and Technologies University of Ioannina Ioannina Greece; ^4^ Microbiology Department, School of Health Science, Faculty of Medicine University of Ioannina Ioannina Greece

**Keywords:** chitosan nanoparticles, dapagliflozin, inflammation, reactive oxygen species, SGLT2i, signaling pathways

## Abstract

Dapagliflozin is a sodium‐glucose cotransporter‐2 (SGLT‐2) inhibitor primarily used to treat type 2 diabetes by lowering blood glucose levels. In addition to its antidiabetic action, it has demonstrated cardioprotective and renoprotective effects, along with antioxidant, anti‐inflammatory, and anticancer activities. Encapsulation of dapagliflozin in nanocarriers represents an innovative strategy to improve existing therapies and develop targeted treatments. Such formulations can enhance solubility and stability, improve epithelial permeability and bioavailability, and reduce potential side effects. This study investigates the cellular and molecular effects of dapagliflozin encapsulated in chitosan nanoparticles, focusing on colon (Caco‐2) cells. The findings showed that free dapagliflozin significantly reduced cell viability, whereas the encapsulated form preserved high cell viability. In addition, the encapsulated drug effectively reduced reactive oxygen species levels, maintaining its antioxidant activity. Free dapagliflozin did not increase the expression of Nrf2, NFκB, or HO‐1 signaling pathways. In contrast, encapsulation in chitosan nanoparticles resulted in increased expression of all three pathways, indicating potential regulatory involvement in these signaling mechanisms.

## Graphical abstract

Dapagliflozin, a SGLT2 inhibitor with antioxidant and anti‐inflammatory properties, was encapsulated in chitosan nanoparticles to enhance targeted delivery to intestinal cells. In Caco‐2 cells, the encapsulated form preserved cell viability, reduced reactive oxygen species, and activated protective pathways (Nrf2, NFκB, HO‐1) more effectively than the free drug. These results highlight chitosan's role as a promising carrier and suggest SGLT2 inhibitors like dapagliflozin could offer new treatments for gut inflammation.

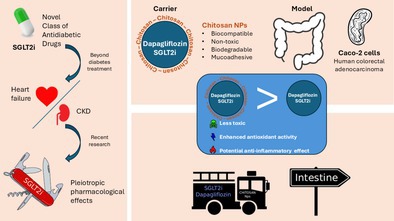

## 1 Background

Nanotechnology, the science of manipulating matter at the nanoscale (1–100 nm), enables the development of novel materials and devices with unique properties that have transformative potential across various fields, including medicine, electronics, and energy [1]. In biomedical research, nanotechnology facilitates the design of nanostructured systems that improve diagnostics, drug delivery, and therapeutic efficacy through advanced techniques such as nanofabrication and high‐resolution microscopy [2]. For instance, the use of nanoparticles enhances the solubility, stability, and bioavailability of bioactive compounds via encapsulation and specialized formulations [3]. Among these, chitosan nanoparticles (CNPs) are extensively employed due to their favorable biocompatibility, biodegradability, and hydrophilicity. CNPs serve as efficient carriers for drug and gene delivery, enabling targeted tumor treatment and improved therapeutic outcomes across various administration routes [4].

SGLT‐2 inhibitors (SGLT2i), a group of drugs mainly prescribed for treating type 2 diabetes mellitus (T2DM), offer notable advantages beyond lowering blood sugar levels, particularly in safeguarding cardiovascular and kidney health [5]. SGLT2i act by inhibiting the reabsorption of glucose and sodium in the kidneys. From 2013 to 2017, the Food and Drug Administration (FDA) approved four SGLT2i (canagliflozin, dapagliflozin, empagliflozin, and ertugliflozin) for managing T2DM. SGLT2i have demonstrated significant reductions in cardiovascular death and heart failure hospitalizations. A series of clinical trials such as EMPA‐REG OUTCOME, CANVAS, EMPEROR‐Reduced, EMPEROR‐Preserved, and SOLOIST‐WHF have shown these benefits in both diabetic and non‐diabetic populations [6, 7, 8, 9, 10].

Recently, the scientific community, through in vitro experiments and preclinical in vivo studies, has focused its attention on exploring additional potential benefits and effects of SGLT2i. Dapagliflozin is the second SGLT2i to receive FDA approval, granted in 2014, 1 year after canagliflozin. Initially, it was approved for patients with T2DM, but it is now the only one among the four available SGLT2i also indicated for patients with cardiovascular and kidney diseases. Specifically, in 2020, dapagliflozin was approved for the treatment of heart failure with reduced ejection fraction in adults, both with and without T2DM. A year later, it was also approved for patients with CKD, aiming to reduce the risk of disease progression [11, 12]. Research interest in dapagliflozin has increased significantly in recent times, driven by ongoing scientific advancements and its diverse therapeutic effects. Current studies are investigating its mechanisms of action in modulating inflammation, oxidative stress, and cellular apoptosis, as well as evaluating its potential benefits in non‐diabetic populations. As a result, dapagliflozin is increasingly recognized for its expanding role in modern therapeutic strategies.

The integration of nanoparticles (NPs) with SGLT2i represents a promising approach in nanomedicine, aiming to enhance therapeutic efficacy and reduce adverse effects in the management of T2DM. NPs offer the ability to act as drug carriers, improving the pharmacokinetic profiles of these agents by enhancing solubility, stability, epithelial permeability, and bioavailability, while also prolonging half‐life and reducing toxicity. The aim of this study was to investigate the cellular and molecular effects of dapagliflozin encapsulated in chitosan nanoparticles (CNPs), with a particular focus on cytocompatibility, epithelial response, and the modulation of key signaling pathways in colon epithelial cells.

## 2 Methods

### 2.1 Synthesis of Chitosan Nanoparticles With or Without Encapsulated Dapagliflozin

Chitosan nanoparticles were synthesized using the ionic gelation method with either sodium tripolyphosphate (TPP) or sodium citrate (SC) as the crosslinker. In the former case, the nanoparticles were formed as described in Bellou et al. [13]. Briefly, a 0.2% w/v chitosan solution, adjusted to pH 5, was used as the stock solution. Blank chitosan nanoparticles were formed by the dropwise addition of the TPP solution (2.5 mg/mL in ultrapure water) to the chitosan stock solution at a CS:TPP weight ratio of 2:1, to a final volume of 1.4 mL. The CS‐TPP solution was then incubated at 1000 rpm and 30°C for 10 min. The mixture was centrifuged at 14,000 rpm for 20 min; the supernatant was discarded, and the precipitate was washed twice with ultrapure water and finally lyophilized.

For the synthesis of chitosan nanoparticles encapsulating Dapagliflozin, the methodology described above was followed with slight modifications. First, Dapagliflozin (10 mM in DMSO) was added to the stock chitosan solution (0.2% w/v) to achieve a final Dapagliflozin concentration of 0.1 mM in a total volume of 1.4 mL. The CS–Dapagliflozin mixture was incubated at 1000 rpm and 30°C for 10 min. The subsequent steps for the addition of TPP were carried out as previously described; however, in this case, the supernatant obtained after centrifugation was retained for the determination of Dapagliflozin encapsulation efficiency.

For the synthesis of chitosan nanoparticles using sodium citrate as the crosslinker, a similar approach was employed [14]. Sodium citrate dihydrate (7.5 mg/mL in ultrapure water) was added dropwise to a 1% w/v chitosan solution (pH 5) at a CS:SC weight ratio of 3.3:1, to a final volume of 1.4 mL. The mixture was incubated at 900 rpm and 25°C for 30 min, centrifuged at 14,000 rpm for 20 min, and washed twice as described above.

For Dapagliflozin encapsulation, the compound (100 mM in DMSO) was added to the chitosan solution to achieve a final concentration of 2.44 mM and incubated at 900 rpm and 25°C for 30 min prior to the addition of sodium citrate. All resulting nanoparticles were stored at 4°C until use.

For the determination of the encapsulation efficiency of Dapagliflozin, the initial absorbance of Dapagliflozin solution (0.1 mM and 2.44 mM for the CS‐TPP and CS‐SC nanoparticles, respectively) and the absorbance of the respective supernatants (final absorbance) were measured spectrophotometrically at 275 nm. The encapsulation efficiency (EE %) of Dapagliflozin was then calculated as presented in Equation (1).

(1) $$\mathrm{EE}\left(\%\right)=\frac{\text{initial absorbance}-\text{final absorbance}}{\text{initial absorbance}}\times 100$$

### 2.2 Cells

The Caco‐2 cells (an adherent cell line isolated from colon tissue derived from a patient with colorectal adenocarcinoma, ATCC‐HTB‐37) were cultured in a nutrient medium without sodium pyruvate (Sigma‐Aldrich D5796). Cells were maintained in Dulbecco's Modified Eagle's Medium (DMEM) supplemented with 10% fetal bovine serum (FBS), 1% L‐glutamine, and 1% penicillin–streptomycin under standard culture conditions (37°C, 5% CO_2_).

The Caco‐2 cells were used as a validated in vitro model of the intestinal epithelial barrier [15] to assess the cytocompatibility of dapagliflozin formulations.

### 2.3 Cell Viability‐ Cytotoxicity Assay

Cell viability was assessed using the MTT assay. Caco‐2 cells were seeded in 96‐well plates (1 × 10^4^ cells/well), incubated for 24 h and then treated with various concentrations of dapagliflozin and the formulations for 24 or 48 h. Control groups were treated with equivalent volumes of dimethyl sulfoxide (DMSO) corresponding to each dapagliflozin concentration. 3‐(4,5‐dimethylthiazol‐2‐yl)‐2,5‐diphenyltetrazolium bromide solution (MTT—stock solution of 3 mg/mL) was added and incubated for 3 h at 37°C. Subsequently, the supernatant was carefully removed, and dimethyl sulfoxide (DMSO) was added to dissolve the formazan crystals. Absorbance was measured at 540 nm (quantification) and 690 nm (background correction) using a spectrophotometer (Infinite 200 Pro, Tecan, Switzerland). Cell viability above 80% was considered non‐cytotoxic, following ISO 10993‐5: 2009 [16].

### 2.4 Clonogenic Assay

To ensure optimal cell integrity and function, cells were seeded in 6‐well plates at a density of 1 × 10^3^ cells per well, incubated for 24 h, and then exposed to different concentrations of dapagliflozin and the formulations for an additional 24 h. Control groups were treated with equivalent volumes of DMSO corresponding to each dapagliflozin concentration. Following treatment, the supernatant was discarded, and fresh culture medium was added. Cells were maintained for 12 days with regular monitoring and medium changes. Colonies were fixed and stained with 6% (v/v) glutaraldehyde (25%) and 0.5% (w/v) crystal violet, then counted to determine the survival fraction (SF) [17].

### 2.5 Flow Cytometry

#### 2.5.1 Reactive Oxygen Species (ROS) Formation

Caco‐2 cells were seeded in 6‐well plates (150 × 10^3^ cells/well) and incubated for 3 h to allow adhesion. Cells were then treated with dapagliflozin and the formulations and incubated for 24 h. After incubation, the supernatant was removed, and cells were washed once with Phosphate Buffered Saline (PBS). Trypsin was added for detachment, and the cells were collected in PBS and centrifuged (3000 rpm, 5 min). The pellet was resuspended in cold Hanks' Balanced Salt Solution (HBSS). For ROS detection, cells were incubated in the dark with 2′,7′‐Dichlorofluorescin diacetate (DCFDA, 2.5 μM). Where applicable, H_2_O_2_ (0.1 mM) was added to induce ROS production. After 30 min, propidium iodide (PI) (Biolegend 421,301) (1 mg/mL) was added, and samples were kept on ice until the flow cytometry analysis (Partec ML, Partec GmbH, Germany).

#### 2.5.2 Apoptosis/Necrosis Assay

Caco‐2 cells were seeded in 12‐well plates (5 × 10^4^ cells/well) and incubated for 24 h before treatment with dapagliflozin and the formulations for an additional 24 h. The next day, the supernatant was collected, and wells were washed with PBS, which was also collected. Cells were detached using trypsin and collected with PBS. Cell counting was performed to ensure each sample contained exactly 1 × 10^5^ cells. Samples were centrifuged (3000 rpm, 5 min), and the supernatant was discarded. The pellet was resuspended in 100 μL Annexin V Binding Buffer (Biolegend 422201). Annexin V (Biolegend 640906) and PI (Biolegend 421301) (5 μg/mL each) were added under dark conditions, and samples were gently vortexed and incubated at room temperature for 15 min in the dark. Before flow cytometry analysis, 500 μL Annexin V Binding Buffer and 400 μL PBS were added. Samples were kept on ice until flow cytometry analysis (Partec ML, Partec GmbH, Germany).

#### 2.5.3 Cell Cycle Assay

Caco‐2 cells were seeded in 6‐well plates (100 × 10^3^ cells/well) and incubated for 24 h before treatment with dapagliflozin and the formulations for an additional 24 h. After treatment, the supernatant was removed, and cells were washed twice with PBS. Cells were detached with trypsin, collected in PBS, and centrifuged (3000 rpm, 5 min). The pellet was resuspended in ice‐cold PBS, centrifuged again, and then fixed by the dropwise addition of absolute ethanol while vortexing. Samples were stored at −20°C for at least 7 days. On the analysis day, cells were centrifuged, resuspended in PBS (950 μL), and incubated with PI and RNAse (25 μg/mL) for 30 min in the dark. Samples were placed on ice until flow cytometry analysis (Partec ML, Partec GmbH, Germany).

### 2.6 Western Blotting

Caco‐2 cells (7.5 × 10^5^) were seeded in Petri dishes, treated with dapagliflozin and the formulations for 24 h, and washed with PBS. Cells were scraped, collected on ice, and lysed in RIPA buffer supplemented with protease and phosphatase inhibitors. Lysates were sonicated and centrifuged (14,800 rpm, 20 min, 4°C), and protein concentration was determined using the BCA Protein Assay Kit (Thermo Fisher Scientific). Equal amounts of protein (20–30 μg) were mixed with Laemmli buffer, denatured at 100°C for 4 min, and separated by SDS‐PAGE (180 V, 2 h). Proteins were transferred into PVDF membranes via wet transfer (200 mA, 90 min, 4°C), stained with ponceau S, washed, and blocked with 5% BSA in tris‐buffered saline with Tween 20 (TBST) (90 min). Membranes were incubated overnight at 4°C with primary antibodies against NF‐κB (Cell Signaling Technology 8242), HO‐1 (Cell Signaling Technology 43,966), NRF2 (Cell Signaling Technology 12,721), and α‐Tubulin (Santa Cruz Biotechnology sc‐5286). After washing, HRP‐conjugated secondary antibody (Cell Signaling Technology 7074) was applied for 1 h at room temperature. Protein bands were detected using the Enhanced Chemiluminescence (ECL) method (Bio‐Rad 1705061), and signal intensity was analyzed using ImageLab software with the ChemiDoc MP Imaging System.

### 2.7 Statistical Analysis

All data are presented as mean ± standard deviation. Statistical significance between mean values was assessed using the Student's *t*‐test. A *p*‐value of less than 0.05 was considered statistically significant.

## 3 Results

### 3.1 Encapsulation of Dapagliflozin to Chitosan Nanoparticles

For the Chitosan Nanoparticles Encapsulating Dapagliflozin (CNPsDapa), the encapsulation efficiency of dapagliflozin was 35% and for the Chitosan Nanoparticles with Tripolyphosphate Encapsulating Dapagliflozin (TPPCNPsDapa), where tripolyphosphate (TPP) acts as a crosslinking agent, the encapsulation efficiency of dapagliflozin was 23%.

### 3.2 Cell Viability

Acetic acid (1% w/v) was used as the solvent for all nanoparticles. Prior to assessing nanoparticle cytotoxicity, its toxicity was evaluated in Caco‐2 cells, revealing viability above 80% after 24 and 48 h, indicating minimal solvent‐induced toxicity.

**FIGURE 1:**
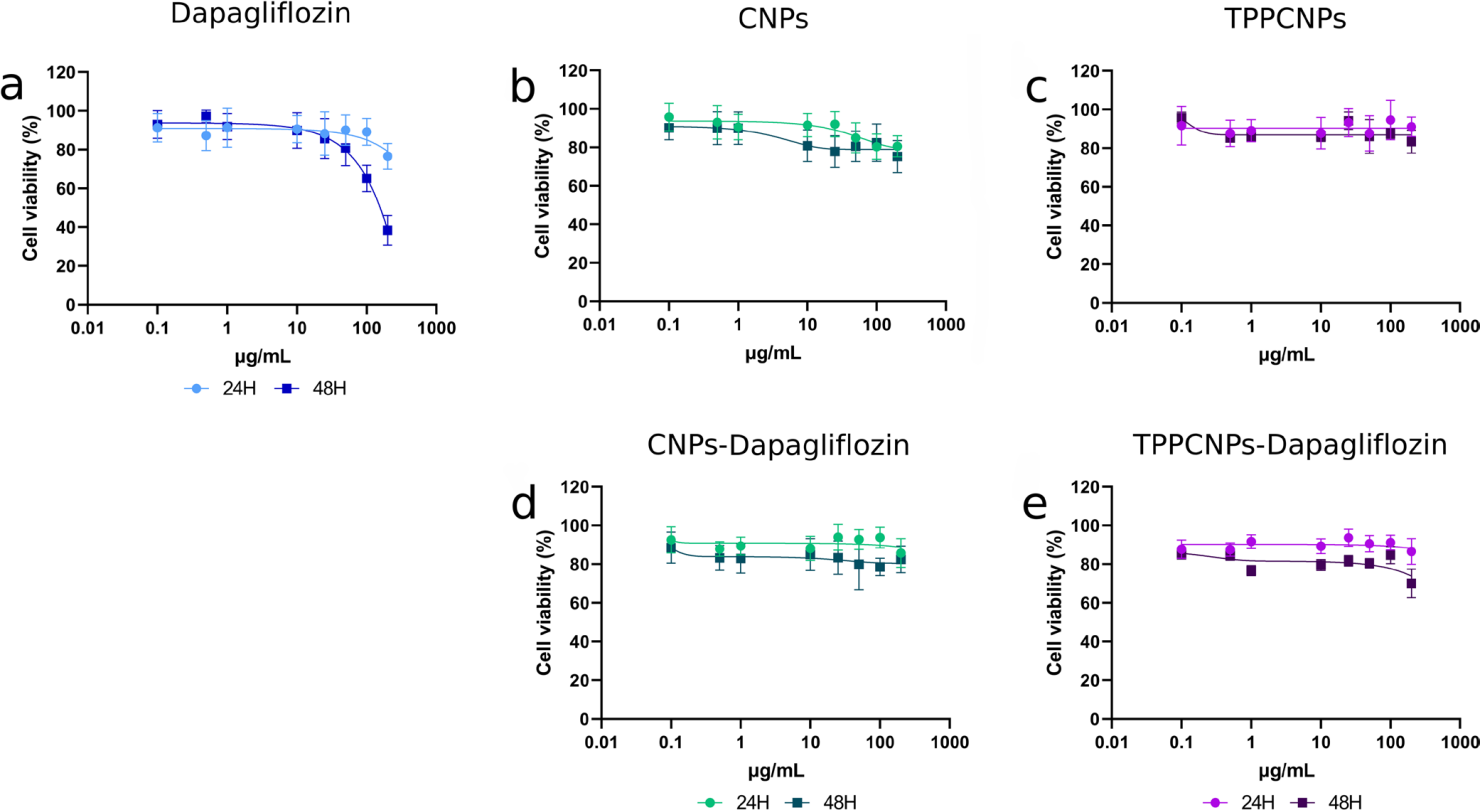
Effect of increasing concentrations of different treatments on the viability of Caco‐2 cells following 24‐ and 48‐h incubation, as determined by the MTT assay. The individual panels correspond to specific treatment groups: (a) dapagliflozin, (b) CNPs, (c) TPPCNPs, (d) CNPs–dapagliflozin, and (e) TPPCNPs–dapagliflozin, as indicated in each graph. Cell viability is expressed as a percentage relative to untreated control cells. Data are presented as mean ± standard deviation from independent experiments.

Free dapagliflozin exhibited dose‐ and time‐dependent cytotoxicity (Figure 1a). After 24 h, viability at 200 μg/mL was 76%, while after 48 h, a decline was observed from 50 μg/mL onward, reaching 65% and 38% at 100 and 200 μg/mL, respectively. In contrast, CNPs had a negligible effect on cell viability (Figure 1b), with survival exceeding 80% across all conditions except at 200 μg/mL, where it declined to 75%. Similarly, TPPCNPs (Figure 1c) showed no significant cytotoxicity, maintaining viability above 80% at all tested concentrations. Encapsulation of dapagliflozin in CNPs and TPPCNPs altered its cytotoxic profile. Both formulations (Figure 1d,e) showed no significant dose‐ or time‐dependent toxicity, with viability remaining ~80% in most conditions. Only TPPCNPsDapa at 200 μg/mL reduced viability to 70% after 48 h.

### 3.3 Assessment of the Colony‐Forming Ability of Caco‐2 Cells

The clonogenic assay evaluates the long‐term cytotoxic effects of tested substances by assessing their impact on cell proliferation. Chitosan nanoparticles were tested at 10, 50, and 100 μg/mL, while dapagliflozin was assessed at 1, 10, and 100 μg/mL.

**FIGURE 2:**
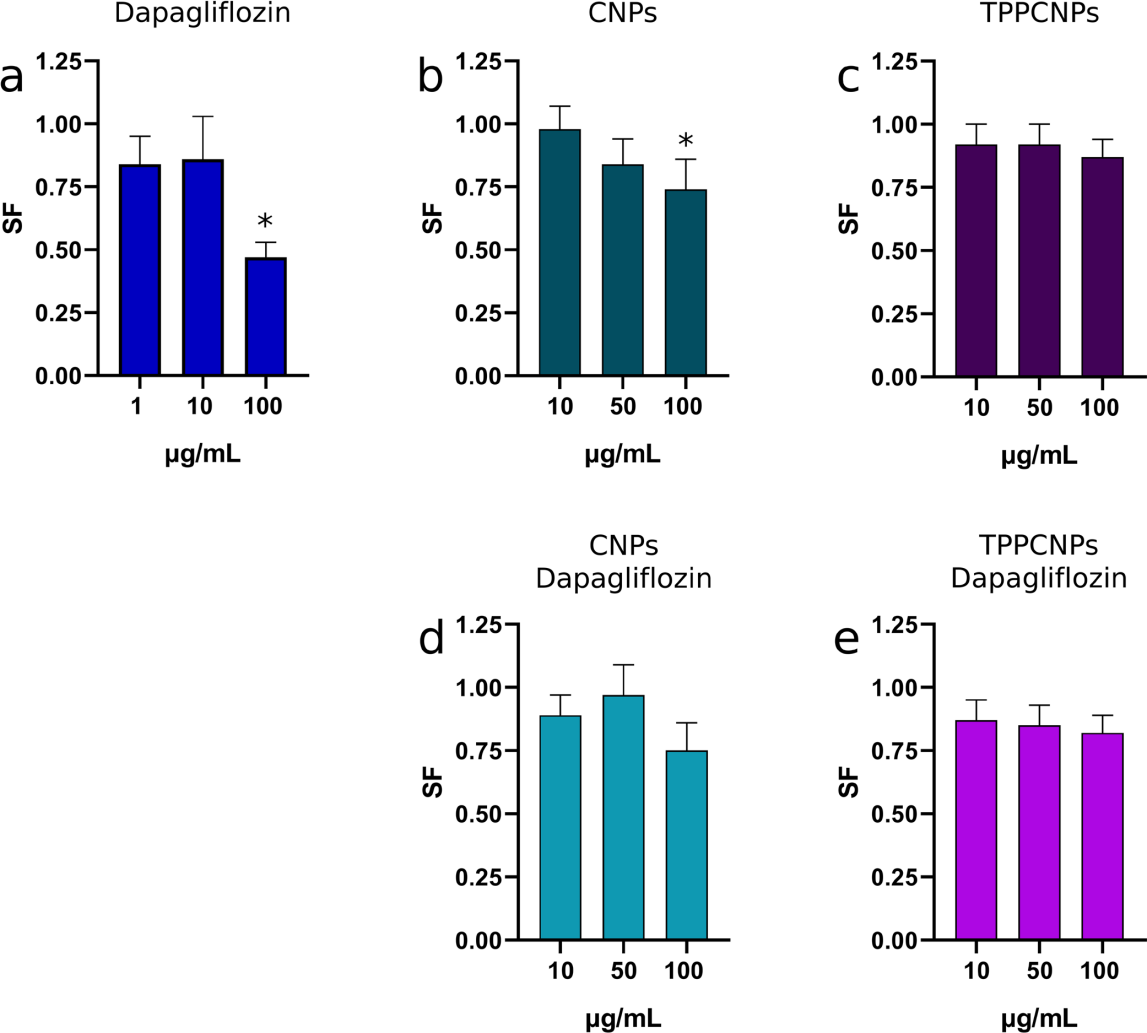
Survival fraction (SF) of Caco‐2 cells after 24‐h exposure to increasing concentrations of the tested formulations. Each panel represents a distinct treatment group: (a) dapagliflozin, (b) CNPs, (c) TPPCNPs, (d) CNPs–dapagliflozin, and (e) TPPCNPs–dapagliflozin, as labeled within the corresponding graphs. The survival fraction is plotted against concentration to illustrate dose‐dependent effects across treatments. Representative images of colony formation for each condition are shown on the right. Data are presented as mean ± standard deviation from independent experiments. *p < 0.05 compared to the control group.

Dapagliflozin significantly reduced the survival fraction only at 100 μg/mL, decreasing viability by 50%, whereas lower concentrations had no effect (Figure 2a). These findings align with MTT results, suggesting irreversible damage at higher concentrations.

CNPs significantly reduced colony formation in a dose‐dependent manner, with a marked decrease at 100 μg/mL (Figure 2b). Interestingly, while the MTT assay suggested minimal short‐term toxicity, the colony formation assay revealed irreversible damage affecting long‐term viability. In contrast, TPPCNPs had no impact on clonogenic potential across all concentrations (Figure 2c), suggesting superior biocompatibility compared to CNPs.

Encapsulation of dapagliflozin within CNPs (CNPsDapa) mitigated cytotoxicity, with no significant reduction in colony formation (Figure 2d). Notably, TPPCNPsDapa also maintained stable survival fractions across all tested concentrations, confirming its biocompatibility (Figure 2e).

### 3.4 Reactive Oxygen Species (ROS) Formation

**FIGURE 3:**
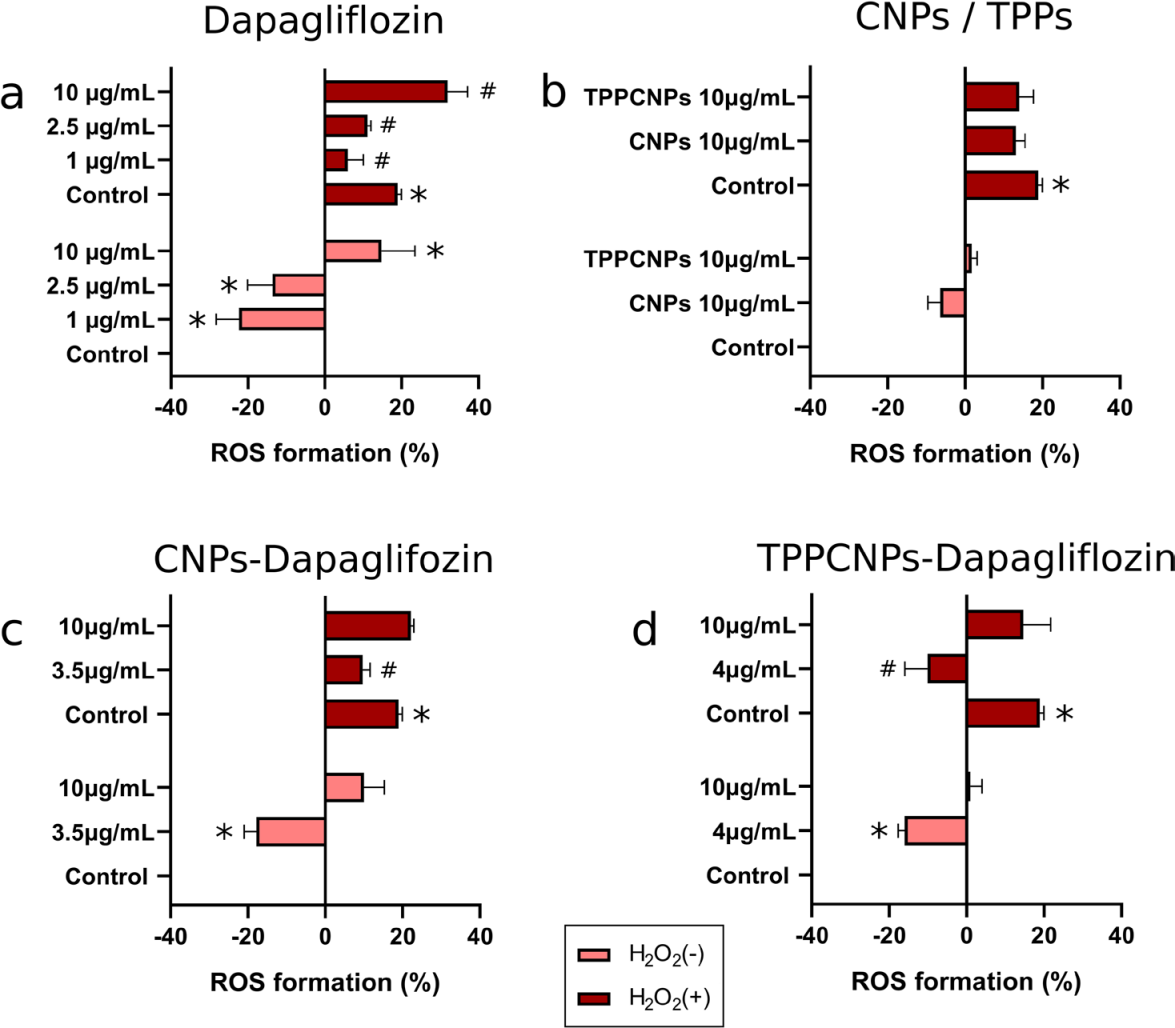
ROS formation (%) for dapagliflozin (a), chitosan carriers (CNPs and TPPCNPs) (b), CNPsDapa (c), and TPPCNPsDapa (d) after 24‐h exposure at different concentrations corresponding to the percentage of encapsulated dapagliflozin in CNPsDapa (~23%) and TPPCNPsDapa (~35%). Data are presented as mean ± standard deviation. *Significant difference compared to the negative control (−H_2_O_2_) (*p* < 0.05). ^#^Significant difference compared to the positive control (+H_2_O_2_) (*p* < 0.05).

Reactive oxygen species levels were assessed via flow cytometry, using H_2_O_2_ as an oxidative stress inducer. Exposure of Caco‐2 cells to H_2_O_2_ increased ROS levels by almost 20% in the control group (*p* < 0.05) (Figure 3). Free dapagliflozin reduced ROS at 1 and 2.5 μg/mL (by 20% and 15%, respectively—*p* < 0.05), but at 10 μg/mL, ROS levels increased by 15% (*p* < 0.05). Under oxidative stress, dapagliflozin at 1 and 2.5 μg/mL lowered ROS by 12% and 6% (*p* < 0.05), while at 10 μg/mL, ROS increased by 13% (*p* < 0.05) (Figure 3a). CNPs at 10 μg/mL reduced ROS by 6%, while TPPCNPs showed negligible effects (Figure 3b). Under oxidative stress, both nanoparticles led to a 6% ROS reduction compared to the positive control.

CNPsDapa at 3.5 μg/mL reduced ROS by 18% (*p* < 0.05) (Figure 3c) and by 9% under oxidative stress (*p* < 0.05). At 10 μg/mL, ROS increased by 10%, while oxidative stress conditions reduced ROS by only 4%. TPPCNPsDapa at 4 μg/mL decreased ROS by 16% (*p* < 0.05), and by 28% under oxidative stress (*p* < 0.05). At 10 μg/mL, no significant ROS reduction occurred (Figure 3d).

Comparing free and encapsulated dapagliflozin, CNPsDapa (3.5 μg/mL) and TPPCNPsDapa (4 μg/mL) exhibited similar antioxidant activity to free dapagliflozin (1 μg/mL), reducing ROS by 20%. Under oxidative stress, TPPCNPsDapa demonstrated greater antioxidant potential than free dapagliflozin or CNPsDapa, suggesting TPPCNPs may enhance dapagliflozin's antioxidant properties.

### 3.5 Apoptosis and Necrosis Analysis

**FIGURE 4:**
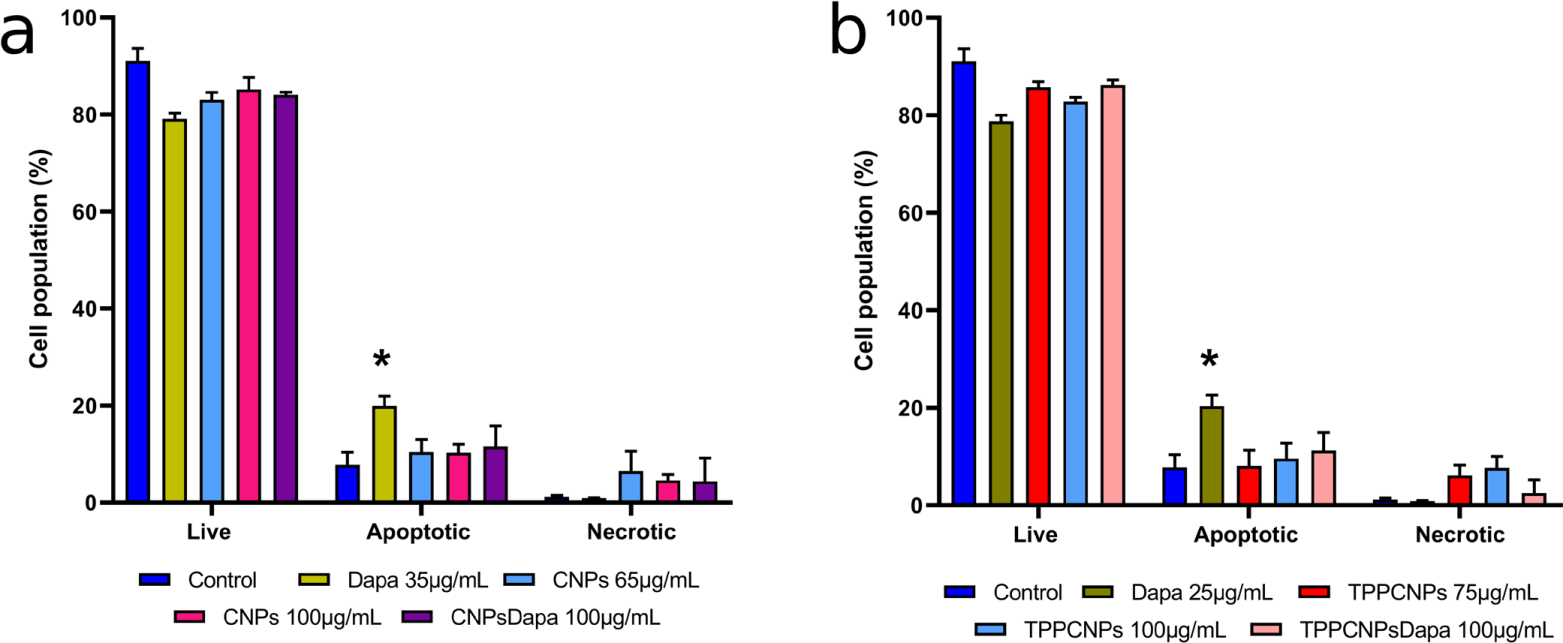
Representation of viable, apoptotic, and necrotic cells (% of the Caco‐2 cell population) following 24‐h incubation with dapagliflozin and CNPsDapa (a) or TPPCNPsDapa (b) at different concentrations corresponding to the percentage of encapsulated dapagliflozin in CNPsDapa (~23%) and TPPCNPsDapa (~35%). Data are presented as mean ± standard deviation. *Significant difference compared to the control (*p* < 0.05).

Flow cytometry assessed apoptosis and necrosis using Annexin V and PI. Free dapagliflozin (35 μg/mL) increased apoptosis by 12% (20% total) without inducing necrosis. CNPs at 65 and 100 μg/mL caused a slight apoptosis increase (3%) and necrosis (5% and 3%). CNPsDapa (100 μg/mL) had minimal impact, resembling CNPs alone (Figure 4a).

TPPCNPs effects were similar to CNPs. At 75 μg/mL, necrosis increased by 6%, while apoptosis remained unchanged. At 100 μg/mL, no dose‐dependent effect was observed. TPPCNPsDapa induced a 5% apoptosis increase and 1% necrosis. Overall, nanoparticle formulations showed no significant apoptotic or necrotic effects compared to free dapagliflozin, which had a greater apoptotic impact (Figure 4b).

### 3.6 Cell Cycle Analysis

**FIGURE 5:**
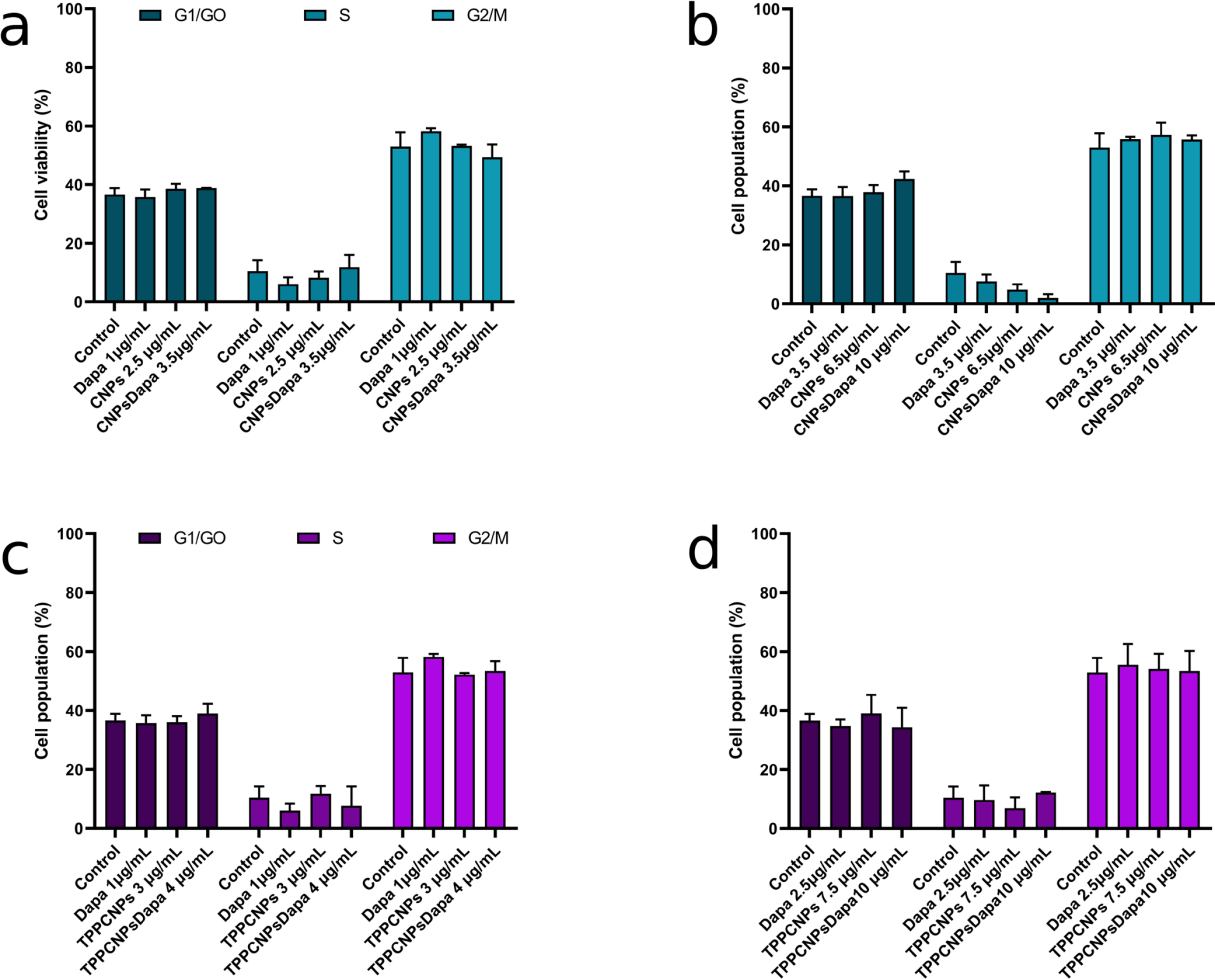
Percentage analysis of the Caco‐2 cell cycle after 24‐h incubation with dapagliflozin and CNPsDapa (a, b) or TPPCNPsDapa (c, d) at different concentrations corresponding to the percentage of encapsulated dapagliflozin in CNPsDapa (~23%) and TPPCNPsDapa (~35%). Data are presented as mean ± standard deviation.

Flow cytometry examined potential cell cycle disruptions. At lower concentrations (Figure 5a), free dapagliflozin (1 μg/mL) reduced the S phase by 4% and increased G2/M by 6%, while CNPs and CNPsDapa had minor effects. At higher concentrations (Figure 5b), CNPsDapa (10 μg/mL) reduced the S phase by 8%, increasing G1/G0 by 6%, suggesting greater cell cycle influence.

TPPCNPs alone had minimal effects (Figure 5c), while TPPCNPsDapa reduced the S phase by 3% and increased G1/G0 by 2%. At higher concentrations (Figure 5d), TPPCNPsDapa caused only minor shifts. No significant cell cycle disruption was observed, except for CNPsDapa at 10 μg/mL, which showed an 8% S phase reduction.

### 3.7 Western Blotting—Signaling Pathway Analysis

**FIGURE 6:**
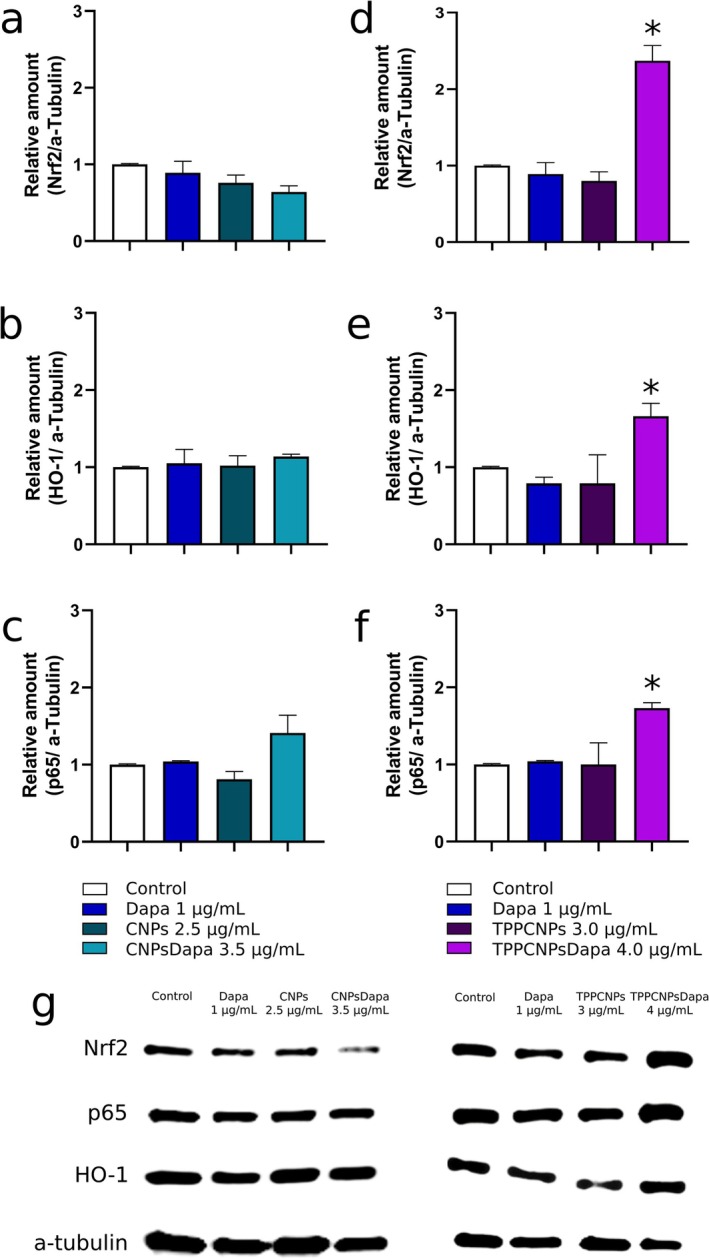
Relative expression density of proteins Nrf2 (a and d), HO‐1 (b and e), p65 (c and f), in comparison to α‐tubulin in Caco‐2 cells after incubation with dapagliflozin and CNPsDapa (a, b and c) or dapagliflozin and TPPCNPsDapa (d, e and f). Representative Western blot images (g). α‐Tubulin was used as a loading control to ensure sample comparability. *Significant difference compared to the control.

Western blotting examined Nrf2, p65, and HO‐1 expression (Figure 6). Nrf2 expression decreased with dapagliflozin, CNPs, and CNPsDapa, lack of activation of the Nrf2 pathway (Figure 6a), consisted with the unchanged HO‐1 levels (Figure 6b). p65 remained stable except for a 1.5‐fold increase with CNPsDapa (Figure 6c). In contrast, TPPCNPsDapa treatment significantly increased Nrf2 (2.4‐fold, *p* < 0.05) (Figure 6d) accompanied by a corresponding increase in HO‐1 by 1.7‐fold (Figure 6e, *p* < 0.05) confirming the activation of the Nrf2‐mediated antioxidant response. Moreover, p65 expression was increased by 1.7‐fold (Figure 6f, *p* < 0.05), indicating modulation of NF‐κB signaling.

## 4 Discussion

The aim of this research was to develop a system for the encapsulation of dapagliflozin and SGLT2i and to study its biological properties in vitro, focusing on cytocompatibility, epithelial response, and modulation of relevant cellular pathways.

Research to date indicates that SGLT2i help reduce inflammation by modulating different signaling pathways. Additionally, they appear to lower oxidative stress, regulate apoptosis—either by promoting or preventing it—and contribute significantly to the maintenance of the body's ionic balance [18].

Dapagliflozin shows anticancer potential by modulating glucose metabolism, apoptosis, and immune responses. It promotes YAP1 degradation in gastric cancer [19], shows selective cytotoxicity in renal cell carcinoma [20] and enhances apoptosis and CD8+ T cell activity in breast cancer models [21]. Also, research indicates that dapagliflozin enhances antioxidant pathways, reduces oxidative stress, and modulates apoptosis, which collectively supports its role in various health conditions. Dapagliflozin activates the Nrf2 antioxidant pathway, leading to increased levels of neurotrophic factors and reduced oxidative stress markers like malondialdehyde (MDA) [22]. In cultured human blood cells, dapagliflozin elevates total antioxidant capacity (TAC) without significantly altering total oxidative stress (TOS) levels, indicating its protective effects against oxidative damage [23].

To enhance the therapeutic efficacy of dapagliflozin, various carrier systems have been explored across different disease models to elucidate its underlying molecular mechanisms. For instance, polymer‐encapsulated dapagliflozin has been shown to attenuate diabetic cataract progression by modulating epithelial‐mesenchymal transition (EMT) pathways [24]. Additionally, co‐delivery with gold nanoparticles has demonstrated renoprotective effects in diabetic nephropathy, mediated through the regulation of specific microRNAs [25]. A mesoporous silica nanoparticle (MSN) system has also been developed for the targeted delivery of dapagliflozin in myocardial infarction models, promoting cardiac tissue repair [26].

Due to its biocompatibility, non‐toxicity, and biodegradability, chitosan remains a highly promising polymer for the encapsulation and delivery of active pharmaceutical compounds. In the context of colon‐targeted drug delivery, the cationic nature of chitosan facilitates strong mucoadhesive interactions, enhancing drug retention and absorption at the mucosal surface [27]. Over the years, numerous chitosan‐based delivery systems have been developed, including crosslinked chitosan, chemically modified chitosan particles, chitosan complexes, chitosan‐coated materials, and other advanced formulations [28]. Notably, chitosan nanoparticles have been employed for the delivery of a wide variety of therapeutic agents across multiple categories, including anticancer drugs, antibiotics, antidiabetic agents, gene and vaccine therapies, neurological drugs, and compounds used in wound healing, among others [29]. Studies investigating the role of chitosan as an effective drug delivery system for managing diabetes mellitus have primarily focused on insulin, as well as GLP‐1 receptor agonists and DPP‐4 inhibitors [30]. However, research on chitosan‐based nanoparticles for the delivery of SGLT2 inhibitors (SGLT2i) remains limited, with only a few studies addressing agents such as empagliflozin and canagliflozin. A recent study reported that intrarectal administration of canagliflozin‐loaded chitosan‐hyaluronic acid microspheres alleviated acetic acid‐induced colitis in rats. This effect was attributed to the inhibition of inflammatory markers (IL‐1β, IL‐18) and reduced caspase‐1 cleavage, achieved via AMPK phosphorylation and downregulation of NF‐κB and NLRP3 expression [31]. Encapsulation of empagliflozin into chitosan nanoparticles has yielded promising results, including high entrapment efficiency (83.8% ± 0.002%), substantial drug loading (42.9% ± 0.03%), and a significantly improved in vitro release profile compared to pure empagliflozin solution. Notably, the intestinal permeability was enhanced by 6.2‐fold, suggesting superior uptake [32]. Further, Sinha et al. developed empagliflozin‐loaded chitosan‐alginate nanoparticles incorporated into an orodispersible film. The formulation exhibited greater cytotoxicity against A549 lung cancer cells than free empagliflozin. In vivo studies revealed higher bioavailability, with increased *C*_max_, *t*_1/2_, and AUC₀–*t* values compared to the free drug. Moreover, the permeability of empagliflozin was enhanced by nearly 7‐fold relative to the reference product, Jardiance [33].

The observed reduction in cytotoxicity of the human colon cell line Caco‐2 following encapsulation should be interpreted primarily as an indication of improved biocompatibility and epithelial tolerance in vitro, rather than a direct enhancement of therapeutic efficacy. Encapsulation reduced the apoptotic effect of dapagliflozin, and the NPs system exhibited antioxidant activity in vitro. The reduction in intracellular ROS, particularly for TPPPCNPsDapa, was consistent with the activation of the Nrf2/HO‐1 pathway observed in Western blot analysis. In contrast, the antioxidant activity of CNPsDapa was not accompanied by an Nrf2 or HO‐1 upregulation that may suggest the involvement of additional Nrf2‐independent antioxidant mechanisms.

The TPPS chitosan‐encapsulated dapagliflozin nanoparticles increased the p65 expression, whereas the CNPs did not. Since no downstream inflammatory mediators were assessed, this increase cannot confirm an anti‐inflammatory response following TPPCNPsDapa treatment. Instead, NF‐κB activation may instead reflect a broader cellular stress response under these experimental conditions, which warrants further investigation. Moreover, protein expression was assessed at a single time point (24 h), which may not fully capture the dynamic regulation of these pathways. As NF‐κB activation is often transient and time‐dependent, earlier or later time points may reveal different patterns of pathway activation. Therefore, the observed changes in p65 expression should be interpreted taking into account the time‐dependent nature of pathway activation.

In conclusion, these findings highlight the potential of chitosan‐based nanoparticle delivery systems to modulate specific cellular signaling pathways. Further investigation into the differential molecular responses may provide insight into targeted therapeutic strategies. Overall, this approach suggests the potential of chitosan‐based nanoparticle systems to improve the in vitro cytocompatibility and intestinal epithelial response to dapagliflozin. However, further in vitro studies on intestinal drug permeability as well as in vivo studies to evaluate gastrointestinal safety and efficacy are required.

## Author Contributions

Conceptualization: Yannis V. Simos, and Haralambos Stamatis. Methodology: Yannis V. Simos and Agni Klonari. Validation: Agni Klonari, Antrea‐Maria Athinodorou and Konstantinos I. Tsamis. Formal analysis: Agni Klonari, Eirini Papanikolaou, Anastasia Skonta, Myrto G. Bellou and Yannis V. Simos; Investigation: Agni Klonari, Eirini Papanikolaou, Antrea‐Maria Athinodorou, Anastasia Skonta, Myrto G. Bellou and Georgios S. Markopoulos. Resources: Dimitrios Peschos and Haralambos Stamatis. Writing – original draft preparation: Agni Klonari, Lampros Lakkas, Petros Bozidis and Konstantinos I. Tsamis. Writing – review and editing: Yannis V. Simos, Lampros Lakkas and Haralambos Stamatis. Visualization: Agni Klonari and Georgios S. Markopoulos. Supervision: Yannis V. Simos. Project administration: Yannis V. Simos. Funding acquisition: Haralambos Stamatis and Dimitrios Peschos. All authors read and approved the final manuscript.

## Funding

The authors have nothing to report.

## Conflicts of Interest

The authors declare no conflicts of interest.

## Supporting information


**Figure S1:** Unprocessed Western blot images used for the figures presented in the main article. The left panel illustrates the effect of free dapagliflozin, CNPs and CNPs dapagliflozin on Nrf2 protein levels in Caco‐2 cells, whereas the right panel illustrates the effect of free dapagliflozin, TPPCNPs and TPPCNPs‐dapagliflozin on Nrf2 protein levels in Caco‐2 cells.
**Figure S2:** Unprocessed Western blot images used for the figures presented in the main article. The right panel illustrates the effect of free dapagliflozin, TPPCNPs and TPPCNPs dapagliflozin on HO‐1 protein levels in Caco‐2 cells.
**Figure S3:** Unprocessed Western blot images used for the figures presented in the main article. The left panel illustrates the effect of free dapagliflozin, CNPs and CNPs dapagliflozin on HO‐1 protein levels in Caco‐2 cells.
**Figure S4:** Unprocessed Western blot images used for the figures presented in the main article. The left panel illustrates the effect of free dapagliflozin, TPPCNPs and TPPCNPs dapagliflozin on p65 protein levels in Caco‐2 cells.
**Figure S5:** Unprocessed Western blot images used for the figures presented in the main article. The left panel illustrates the effect of free dapagliflozin, CNPs and CNPs dapagliflozin on p65 protein levels in Caco‐2 cells.
**Figure S6:** Unprocessed Western blot images used for the figures presented in the main article. The left panel illustrates the effect of free dapagliflozin, CNPs and CNPs dapagliflozin on tubulin protein levels in Caco‐2 cells, whereas the right panel illustrates the effect of free dapagliflozin, TPPCNPs and TPPCNPs‐dapagliflozin on tubulin protein levels in Caco‐2 cells.

## Data Availability

The datasets generated during and/or analyzed during the current study are available from the corresponding author on reasonable request.
