# Supplementary material for: Enhancing the Therapeutic Profile of Dapagliflozin: Chitosan Nanoparticle Encapsulation for Intestinal Applications
Source: FASEB Bioadv. 2026 May 14;8(5):e70116. doi: 10.1096/fba.2026-00050 (PMC13173360; doi:10.1096/fba.2026-00050)
Supplement: Supplementary file 1 — Figure S1: Unprocessed Western blot images used for the figures presented in the main article. The left panel illustrates the effect of free dapagliflozin, CNPs and CNPs dapagliflozin on Nrf2 protein levels in Caco‐2 cells, whereas the right panel illustrates the effect of free dapagliflozin, TPPCNPs and TPPCNPs‐dapagliflozin on Nrf2 protein levels in Caco‐2 cells. Figure S2: Unprocessed Western blot images used for the figures presented in the main article. The right panel illustrates the effect of free dapagliflozin, TPPCNPs and TPPCNPs dapagliflozin on HO‐1 protein levels in Caco‐2 cells. Figure S3: Unprocessed Western blot images used for the figures presented in the main article. The left panel illustrates the effect of free dapagliflozin, CNPs and CNPs dapagliflozin on HO‐1 protein levels in Caco‐2 cells. Figure S4: Unprocessed Western blot images used for the figures presented in the main article. The left panel illustrates the effect of free dapagliflozin, TPPCNPs and TPPCNPs dapagliflozin on p65 protein levels in Caco‐2 cells. Figure S5: Unprocessed Western blot images used for the figures presented in the main article. The left panel illustrates the effect of free dapagliflozin, CNPs and CNPs dapagliflozin on p65 protein levels in Caco‐2 cells. Figure S6: Unprocessed Western blot images used for the figures presented in the main article. The left panel illustrates the effect of free dapagliflozin, CNPs and CNPs dapagliflozin on tubulin protein levels in Caco‐2 cells, whereas the right panel illustrates the effect of free dapagliflozin, TPPCNPs and TPPCNPs‐dapagliflozin on tubulin protein levels in Caco‐2 cells. [file FBA2-8-e70116-s001.pdf]

NRF2

CNPS - TPPCNPs

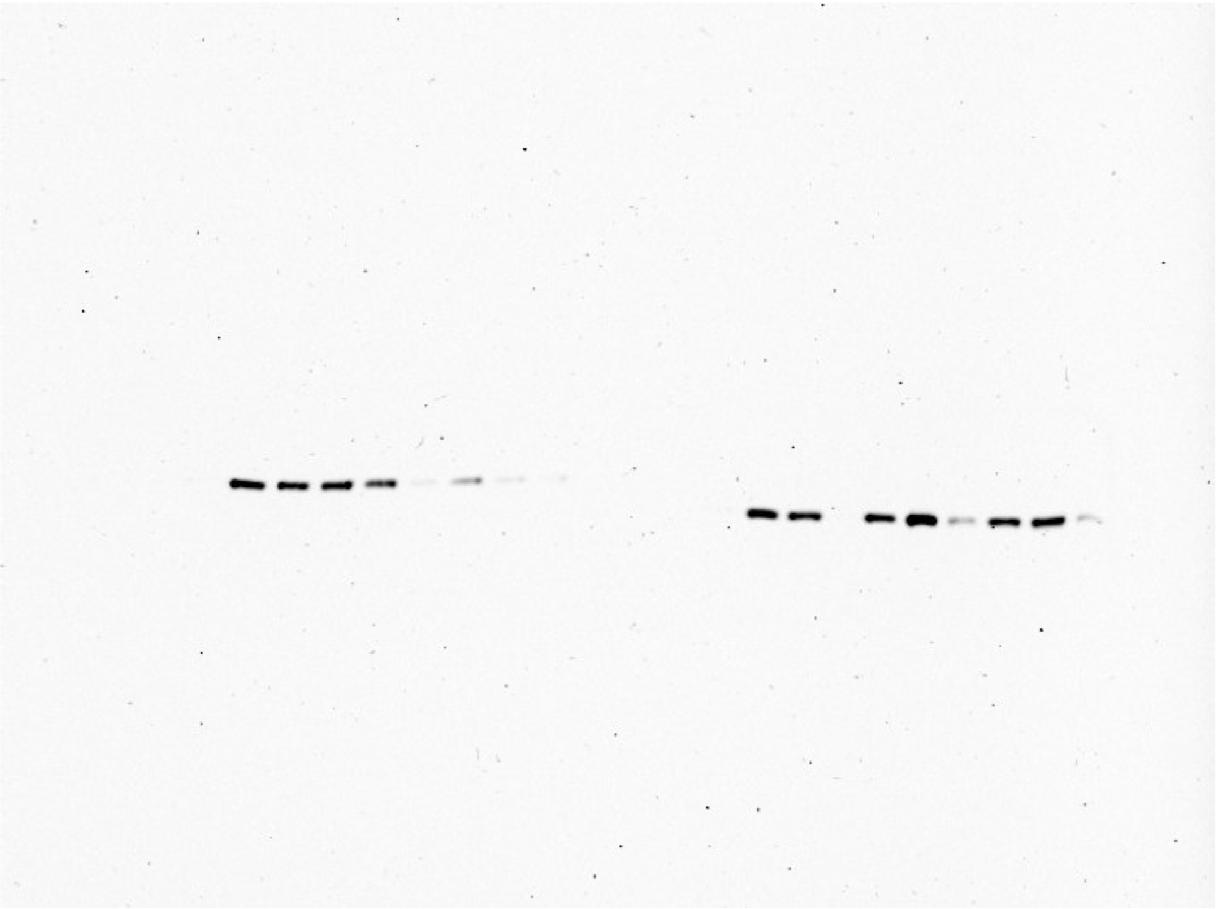

Figure S1. Unprocessed Western blot images used for the figures presented in the main article. The left panel illustrates the effect of free dapagliflozin, CNPs and CNPs-dapagliflozin on Nrf2 protein levels in Caco-2 cells, whereas the right panel illustrates the effect of free dapagliflozin, TPPCNPs and TPPCNPs-dapagliflozin on Nrf2 protein levels in Caco-2 cells.

HO-1

TPPCNPs

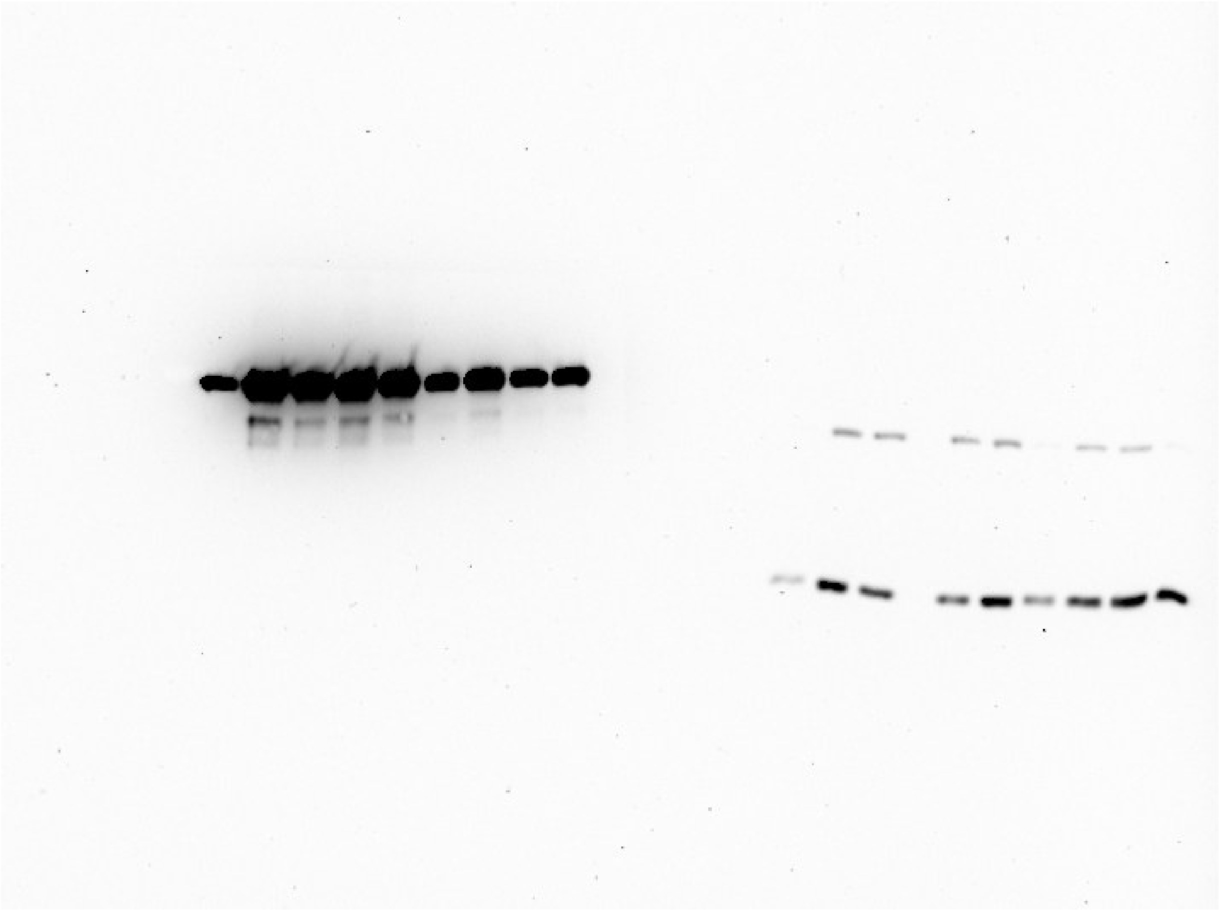

Figure S2. Unprocessed Western blot images used for the figures presented in the main article. The right panel illustrates the effect of free dapagliflozin, TPPCNPs and TPPCNPs-dapagliflozin on HO-1 protein levels in Caco-2 cells.

HO-1

CNPs

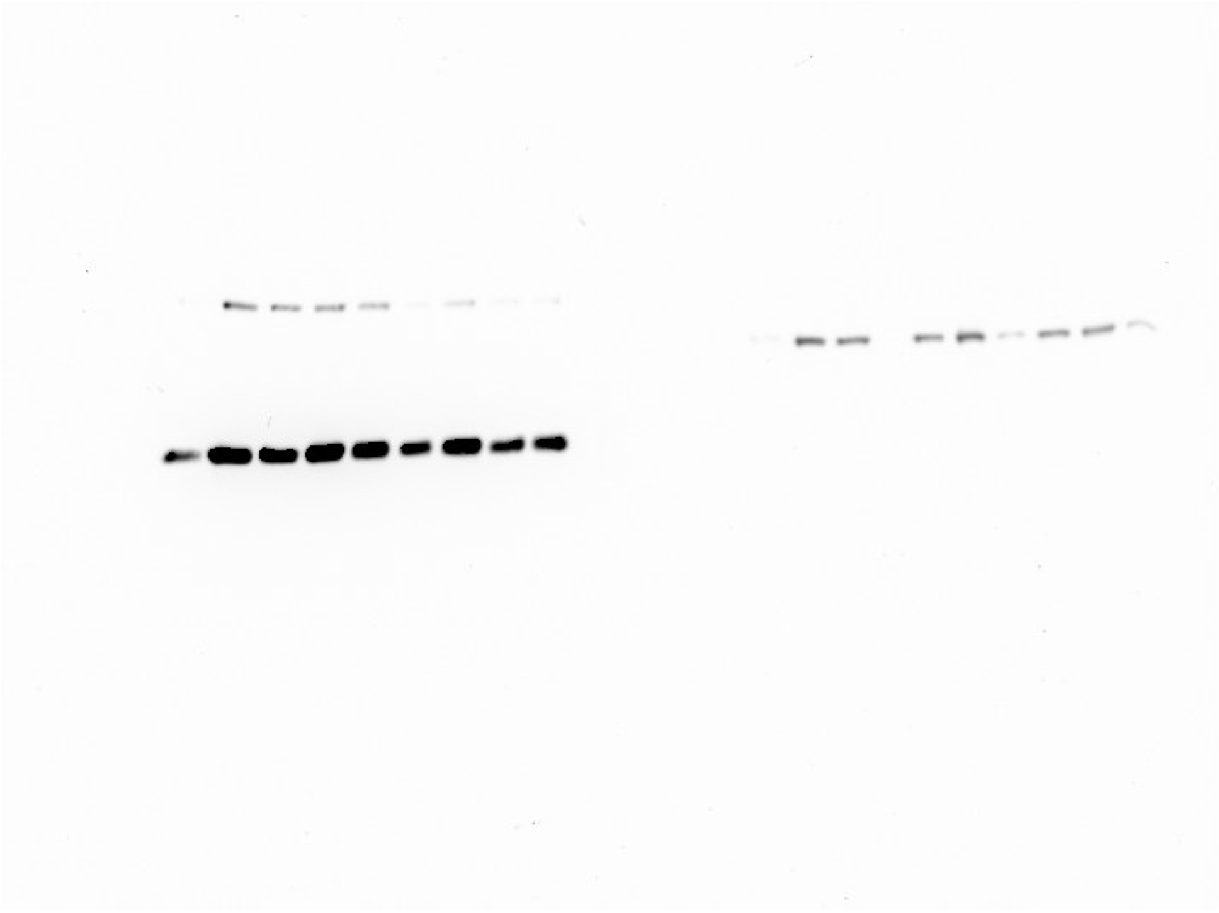

Figure S3. Unprocessed Western blot images used for the figures presented in the main article. The left panel illustrates the effect of free dapagliflozin, CNPs and CNPs-dapagliflozin on HO-1 protein levels in Caco-2 cells.

p65

TPPCNPs

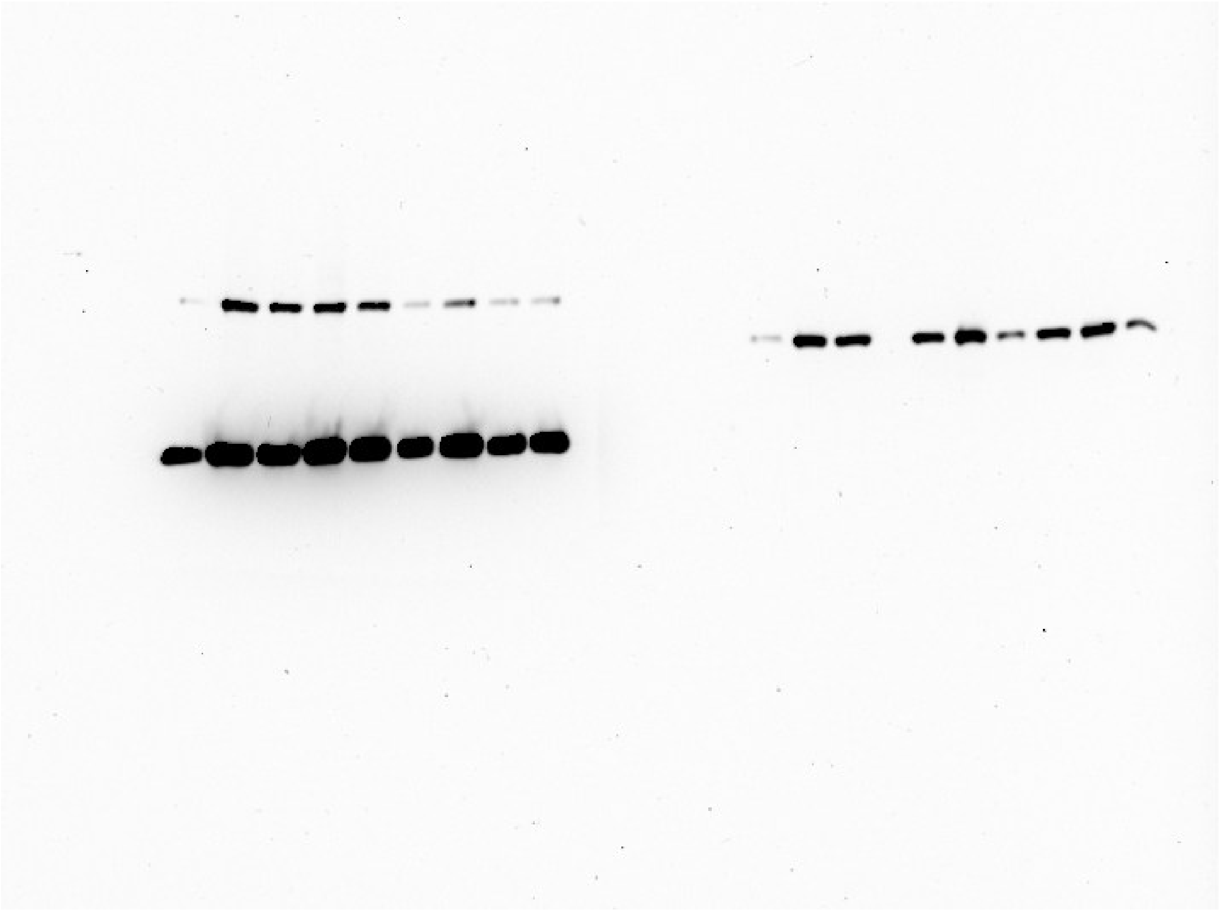

Figure S4. Unprocessed Western blot images used for the figures presented in the main article. The left panel illustrates the effect of free dapagliflozin, TPPCNPs and TPPCNPs-dapagliflozin on p65 protein levels in Caco-2 cells.

p65

CNPs

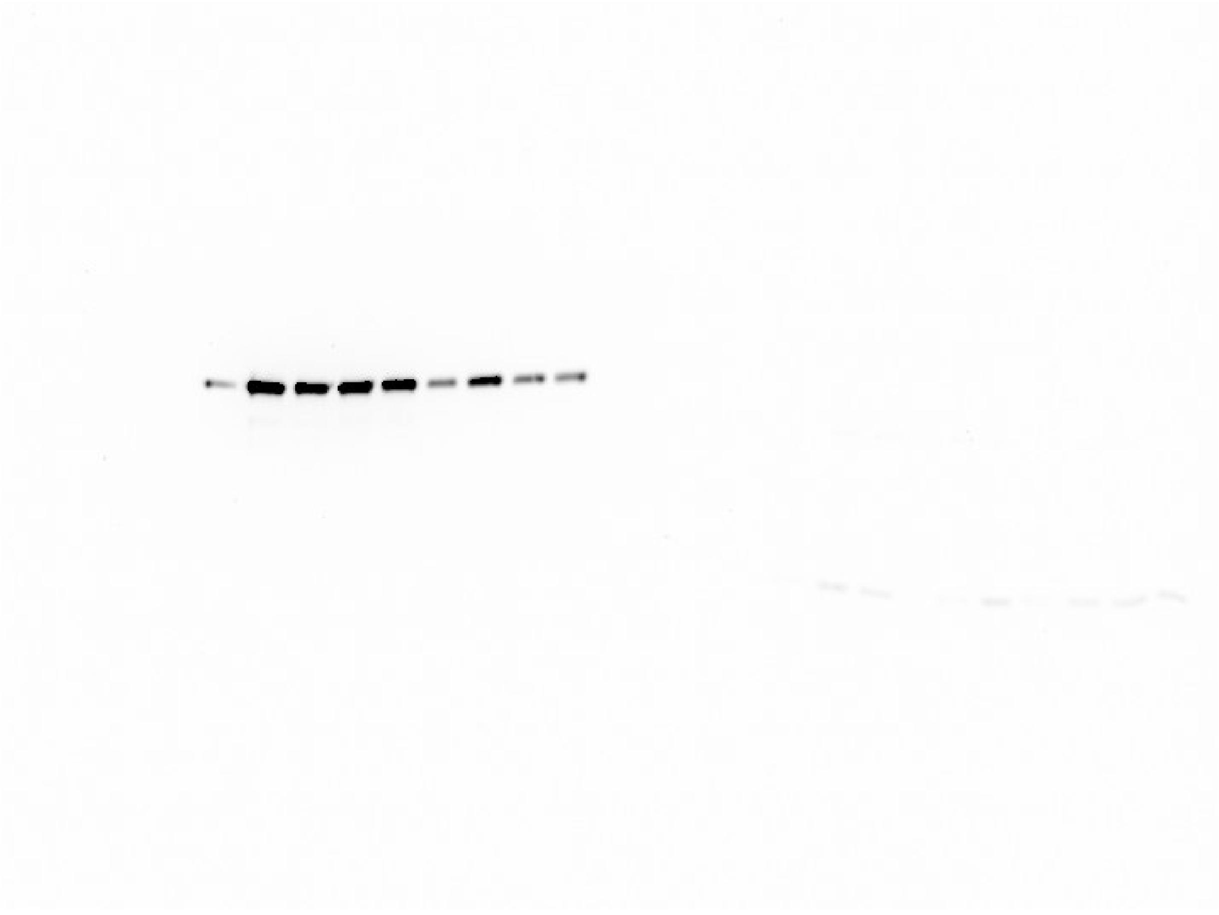

Figure S5. Unprocessed Western blot images used for the figures presented in the main article. The left panel illustrates the effect of free dapagliflozin, CNPs and CNPs-dapagliflozin on p65 protein levels in Caco-2 cells.

Tubulin

CNPs - TPPCNPs

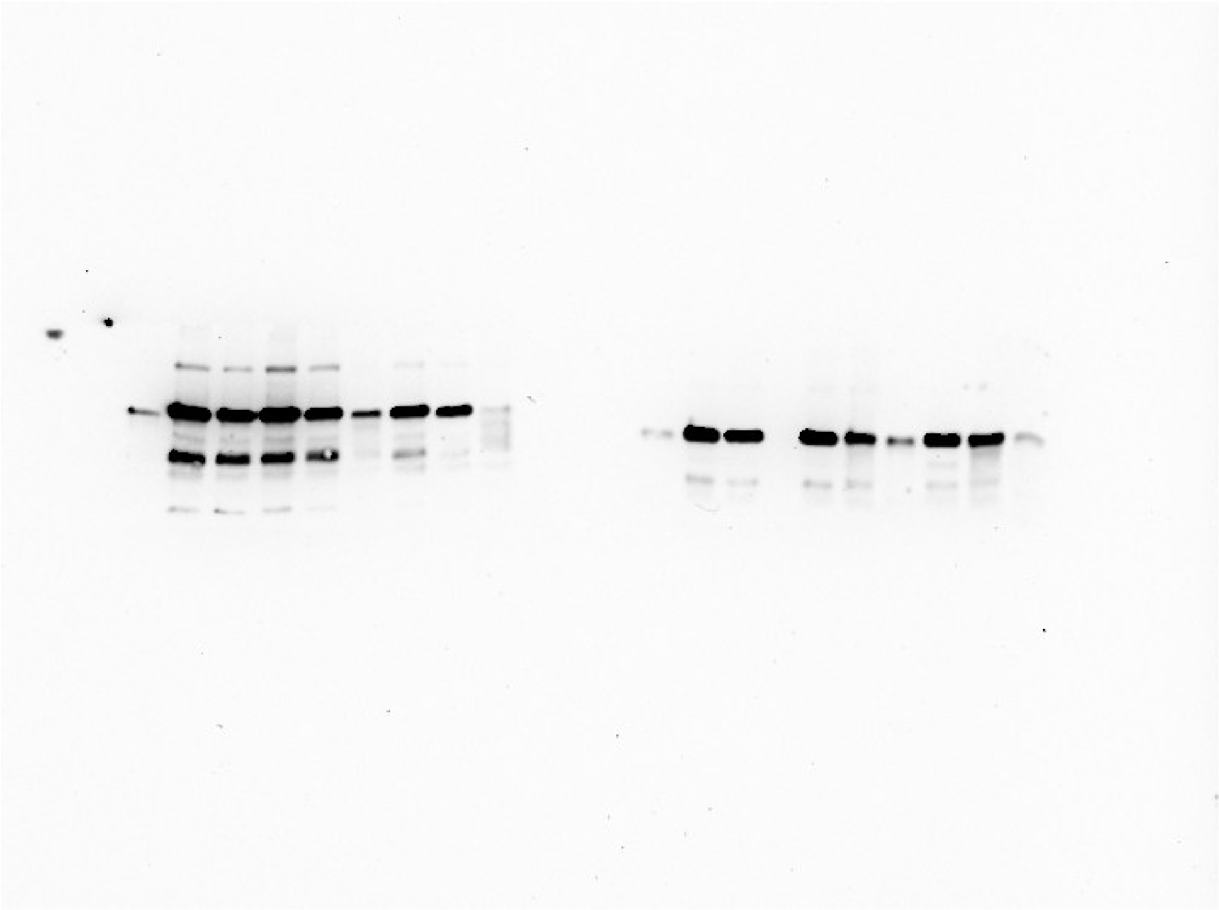

Figure S6. Unprocessed Western blot images used for the figures presented in the main article. The left panel illustrates the effect of free dapagliflozin, CNPs and CNPs-dapagliflozin on tubulin protein levels in Caco-2 cells, whereas the right panel illustrates the effect of free dapagliflozin, TPPCNPs and TPPCNPs-dapagliflozin on tubulin protein levels in Caco-2 cells.

General comment: The original Western blot images display additional bands compared to the final figures because the experiments were performed in duplicate and included a wider range of tested concentrations. Furthermore, the blots include samples of free dapagliflozin as well as dapagliflozin formulated with the corresponding chitosan nanoparticles. Only the conditions relevant to the main findings are presented in the final figures for clarity and conciseness.
